# Bis[4-(4-bromophenylimino-κ*N*)pent-2-en-2-olato-κ*O*]copper(II)

**DOI:** 10.1107/S1600536812042420

**Published:** 2012-10-20

**Authors:** Paul S. E. Bungu, Marietjie Schutte, G. Steyl

**Affiliations:** a Department of Chemistry, University of the Free State, PO Box 339, Nelson Mandela Drive, Bloemfontein 9301, South Africa

## Abstract

In the title compound, [Cu(C_11_H_11_BrNO)_2_], the Cu^II^ atom is in a distorted square-planar geometry, with the two bidentate ketimine ligands positioned in a *trans* geometry. Two inter­molecular C—H⋯O hydrogen bond inter­actions are present which link the mol­ecules in a zigzag manner along the *a* axis. The mol­ecules pack in layers along the diagonal of the *bc* plane.

## Related literature

For similar structures, see: Bourget-Merle *et al.* (2002 ▶); Bryndin *et al.* (2008 ▶) Hsu *et al.* (2004 ▶, 2007 ▶) John *et al.* (2007 ▶); Stender *et al.* (2001 ▶). 
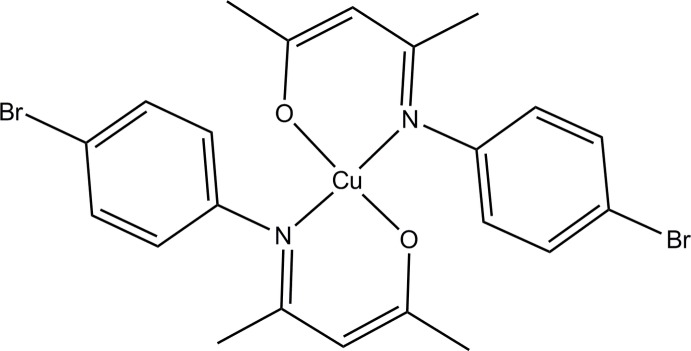


## Experimental

### 

#### Crystal data

[Cu(C_11_H_11_BrNO)_2_]
*M* *_r_* = 569.77
Monoclinic, 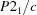

*a* = 12.493 (3) Å
*b* = 11.559 (4) Å
*c* = 15.415 (4) Å
β = 92.306 (14)°
*V* = 2224.2 (11) Å^3^
*Z* = 4
Mo *K*α radiation
μ = 4.60 mm^−1^
*T* = 100 K
0.64 × 0.25 × 0.15 mm

#### Data collection

Bruker APEXII CCD diffractometer
Absorption correction: multi-scan (*SADABS*; Bruker, 2008 ▶) *T*_min_ = 0.262, *T*_max_ = 0.502
13700 measured reflections
5558 independent reflections
4291 reflections with *I* > 2σ(*I*)
*R*_int_ = 0.034

#### Refinement

*R*[*F* ^2^ > 2σ(*F* ^2^)] = 0.034
*wR*(*F* ^2^) = 0.082
*S* = 1.00
5558 reflections
266 parameters
H-atom parameters constrained
Δρ_max_ = 0.65 e Å^−3^
Δρ_min_ = −0.89 e Å^−3^

### 

Data collection: *APEX2* (Bruker, 2008 ▶); cell refinement: *SAINT-Plus* (Bruker, 2008 ▶); data reduction: *SAINT-Plus*; program(s) used to solve structure: *SHELXS97* (Sheldrick, 2008 ▶); program(s) used to refine structure: *SHELXL97* (Sheldrick, 2008 ▶); molecular graphics: *DIAMOND* (Brandenburg & Putz, 2005 ▶); software used to prepare material for publication: *WinGX* (Farrugia, 1999 ▶).

## Supplementary Material

Crystal structure: contains datablock(s) global, I. DOI: 10.1107/S1600536812042420/gg2104sup1.cif

Structure factors: contains datablock(s) I. DOI: 10.1107/S1600536812042420/gg2104Isup2.hkl

Additional supplementary materials:  crystallographic information; 3D view; checkCIF report

## Figures and Tables

**Table 1:** Selected bond lengths (Å)

N1—Cu1	1.958 (2)
N2—Cu1	1.948 (2)
O1—Cu1	1.9110 (17)
O2—Cu1	1.9085 (19)

**Table 2:** Hydrogen-bond geometry (Å, °)

*D*—H⋯*A*	*D*—H	H⋯*A*	*D*⋯*A*	*D*—H⋯*A*
C115—H115⋯O1^i^	0.95	2.47	3.370 (3)	157
C215—H215⋯O2^ii^	0.95	2.54	3.378 (3)	147

Symmetry codes: (i) 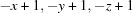; (ii) 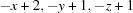.

**Fig. 1.**
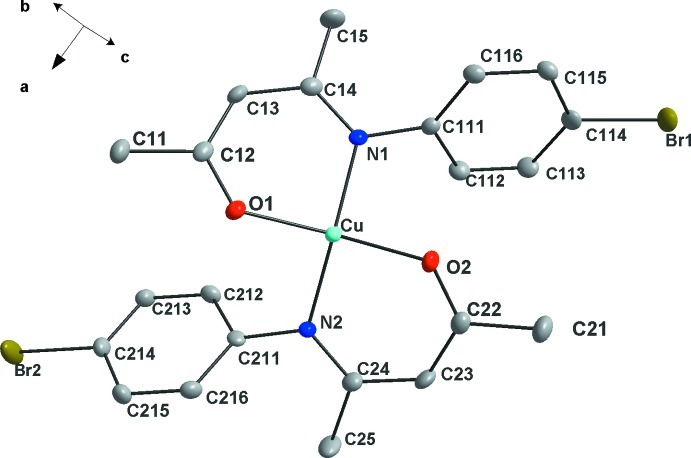
Representation of the title compound, showing the numbering scheme and displacement ellipsoids (50% probability).

**Fig. 2.**
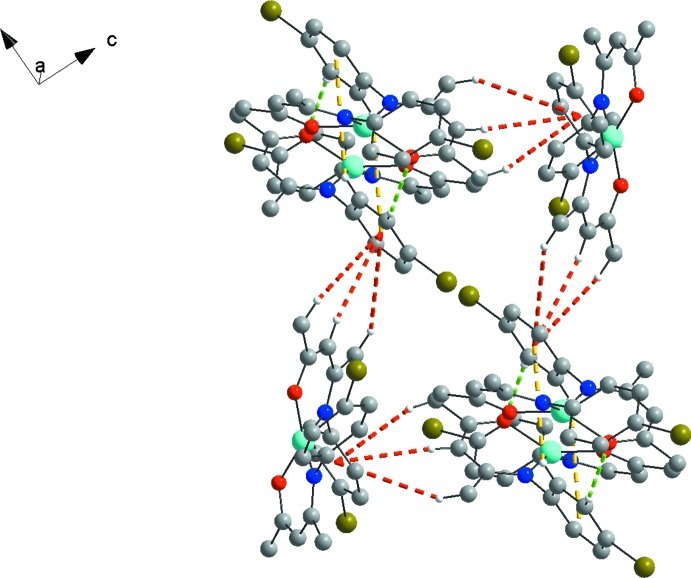
Observed hydrogen interactions (green dashed line), π-π stacking (yellow dashed lines) and π-interactions(red dashed lines) in the crystal structure (hydrogen atoms omitted for clarity).

## supplementary crystallographic information

### Comment

Beta-diketimine ligands are a versatile class of molecules that display an impressive range of diverse applications in coordination chemistry (Bourget-Merle *et al.* 2002). The success of these ligands (Stender *et al.* 2001) is presumably due to the scope for suitable tuning of the steric and electronic properties and due to its easy synthetic accessibility to coordinate to early transition metals (Hsu *et al.* 2004). Here we report the crystal structure of the Cu(II) complex containing a ketiminate ligand, [OC(Me)CHC(Me)NH (Ar)] where Ar = 4-bromophenyl. Structural analysis shows that the title compound crystallized as black cuboidal crystals in the monoclinic space group, *P*2_1_/c, with one molecule in the asymmetric unit and with approximately non-crystallographic *C*2 symmetry. The complex shows a four coordinate environment around the copper atom where two ketimine ligands act as bidentate *N*,*O*-chelators and lie in the *trans* conformation to create two six-membered chelate rings (Cu—O—C—C—C—N). Similar structures are reported in literature and the bond distances and angles of this structure compare well to those in literature (Bryndin *et al.* 2008, Hsu *et al.* 2007, John *et al.*2007).

In this structure, the dihedral angles of the two coordinate planes, O1—Cu1—N2 and O2—Cu1—N1, are 24.1 (2)° and 21.1 (2)° respectively. Also, the Cu—O and Cu—N bond distances are all marginally unequal, hence suggesting distorted square-planer geometry around the Cu(II) center. In Figure 1, the Cu1—O1 and Cu1—O2 bond lengths are 1.911 (2) Å and 1.909 (2) Å while the Cu1—N1 and Cu1—N2 bond lengths were 1.958 (2) Å and 1.948 (2) Å respectively. The presence of the 4-bromophenyl group has caused a slight decrease in the Cu—N bond distances when compared to the respective analogues of copper ketimine complexes by Bryndin *et al.* 2008 (Cu—N = 1.960 (2) Å and 1.965 (2) Å) and Hsu *et al.* 2007 (Cu—N = 1.974 (1) Å and 1.974 (1) Å). Owing to the presence of the distortion, the four N—Cu—O bond angles are marginally different from the ideal value of a square planer geometry of 90° and are reported as 94.70 (8)° (N1—Cu1—O1) and 94.80 (8)° (N2—Cu1—O2). The diagonal angles, N1—Cu1—N2 and O1—Cu1—O2, are reported as 148.20 (9)° and 145.59 (8)° which also differ substantially from the ideal angle of 180° and are comparable to results reported by Bryndin *et al.* 2008 and Hsu *et al.* 2007.These differences were made apparent when comparing the results to the structures by John *et al.* 2007, where phenoxy-ketimine ligands were used as bidentate ligands for both Cu(II) and Ni(II), in which almost a perfect square planar geometry was obeserved. The stabilization is dominated by two intermolecular C—H···O hydrogen bonds (C115—H115···O1) 2.47 Å and C215—H215···O1 = 2.54 Å) (Table 2). π–π stacking is observed between neighbouring molecules with a centroid-to-hydrogen bond distance of 3.7236 (9) Å. Five pi-interactions (centroid-to-hydrogen) are observed with a distance varying between 3.527 (1) Å and 3.746 (1) Å. The hydrogen and pi-interactions as well as the pi–pi stacking is illustrated in Figure 2. The molecules pack in alternating layers along the *c* axis possibly due to the hydrogen- and π interactions.

### Experimental

Copper nitrate Cu(NO_3_)_2_ (100 mg, 0.044 mmol) was dissolved in MeOH and refluxed with 2 equivalent of C_11_H_11_BrONH (237 mg, 0.94 mmol) for 2 h. The product was filtered at ambient temperature and dried in air. Black crystals were grown overnight from chloroform/ether (1:1, 10 ml) mixture. (yield: 169 mg, 0.29 mmol and 67%).

### Refinement

All aromatic and methine H atoms were positioned geometrically and allowed to ride on their parent atoms, with *U*_iso_(H) = 1.2*U*_eq_(C) of the parent atom with the C—H distance of 0.95 Å. The methyl H atoms were placed in geometrically idealized positions and contrained to ride the parent atom with *U*_iso_(H)= 1.5*U*_eq_(C) and at a distance of) 0.98 Å.

### Crystal data

**Table:**

[Cu(C_11_H_11_BrNO)_2_]	*F*(000) = 1132
*M**_r_* = 569.77	*D*_x_ = 1.702 Mg m^−^^3^*D*_m_ = 1.702 Mg m^−^^3^*D*_m_ measured by not measured
Monoclinic, *P*2_1_/*c*	Mo *K*α radiation, λ = 0.71073 Å
Hall symbol: -P 2ybc	Cell parameters from 4022 reflections
*a* = 12.493 (3) Å	θ = 2.6–28.3°
*b* = 11.559 (4) Å	µ = 4.60 mm^−^^1^
*c* = 15.415 (4) Å	*T* = 100 K
β = 92.306 (14)°	Cuboid, black
*V* = 2224.2 (11) Å^3^	0.64 × 0.25 × 0.15 mm
*Z* = 4	

### Data collection

**Table:**

Bruker APEXII CCD diffractometer	4291 reflections with *I* > 2σ(*I*)
Graphite monochromator	*R*_int_ = 0.034
φ and ω scans	θ_max_ = 28.4°, θ_min_ = 3.5°
Absorption correction: multi-scan (*SADABS*; Bruker, 2008)	*h* = −16→16
*T*_min_ = 0.262, *T*_max_ = 0.502	*k* = −15→15
13700 measured reflections	*l* = −20→20
5558 independent reflections	

### Refinement

**Table:**

Refinement on *F*^2^	Primary atom site location: structure-invariant direct methods
Least-squares matrix: full	Secondary atom site location: difference Fourier map
*R*[*F*^2^ > 2σ(*F*^2^)] = 0.034	Hydrogen site location: inferred from neighbouring sites
*wR*(*F*^2^) = 0.082	H-atom parameters constrained
*S* = 1.00	*w* = 1/[σ^2^(*F*_o_^2^) + (0.0411*P*)^2^] where *P* = (*F*_o_^2^ + 2*F*_c_^2^)/3
5558 reflections	(Δ/σ)_max_ = 0.001
266 parameters	Δρ_max_ = 0.65 e Å^−^^3^
0 restraints	Δρ_min_ = −0.89 e Å^−^^3^

### Special details

**Table:**

Geometry. All s.u.'s (except the s.u. in the dihedral angle between two l.s. planes) are estimated using the full covariance matrix. The cell s.u.'s are taken into account individually in the estimation of s.u.'s in distances, angles and torsion angles; correlations between s.u.'s in cell parameters are only used when they are defined by crystal symmetry. An approximate (isotropic) treatment of cell s.u.'s is used for estimating s.u.'s involving l.s. planes.
Refinement. Refinement of *F*^2^ against ALL reflections. The weighted *R*-factor *wR* and goodness of fit *S* are based on *F*^2^, conventional *R*-factors *R* are based on *F*, with *F* set to zero for negative *F*^2^. The threshold expression of *F*^2^ > 2σ(*F*^2^) is used only for calculating *R*-factors(gt) *etc*. and is not relevant to the choice of reflections for refinement. *R*-factors based on *F*^2^ are statistically about twice as large as those based on *F*, and *R*- factors based on ALL data will be even larger.

### Fractional atomic coordinates and isotropic or equivalent isotropic displacement parameters (Å^2^)

**Table:**

	*x*	*y*	*z*	*U*_iso_*/*U*_eq_	
C11	0.8322 (2)	0.8020 (3)	0.36239 (16)	0.0212 (6)	
H11A	0.9055	0.813	0.3859	0.032*	
H11B	0.8011	0.8772	0.3462	0.032*	
H11C	0.8331	0.7522	0.311	0.032*	
C12	0.76552 (18)	0.7460 (2)	0.43032 (15)	0.0149 (5)	
C13	0.66749 (18)	0.7913 (2)	0.44918 (15)	0.0175 (5)	
H13	0.6461	0.8593	0.4185	0.021*	
C14	0.59432 (18)	0.7476 (2)	0.50978 (16)	0.0167 (5)	
C15	0.48851 (19)	0.8093 (3)	0.51301 (18)	0.0243 (6)	
H15A	0.4366	0.7727	0.4723	0.036*	
H15B	0.4978	0.8906	0.4969	0.036*	
H15C	0.4622	0.8048	0.572	0.036*	
C21	0.6951 (2)	0.2875 (3)	0.71567 (18)	0.0256 (6)	
H21A	0.6228	0.3133	0.7287	0.038*	
H21B	0.7318	0.259	0.7689	0.038*	
H21C	0.6905	0.2252	0.6725	0.038*	
C22	0.7570 (2)	0.3872 (2)	0.68015 (17)	0.0179 (5)	
C23	0.8587 (2)	0.4111 (2)	0.71350 (17)	0.0202 (6)	
H23	0.8847	0.3643	0.7604	0.024*	
C24	0.92856 (19)	0.4990 (2)	0.68462 (16)	0.0177 (5)	
C25	1.0380 (2)	0.5049 (3)	0.73005 (18)	0.0294 (7)	
H25A	1.0898	0.463	0.6959	0.044*	
H25B	1.0349	0.4697	0.7877	0.044*	
H25C	1.0601	0.586	0.736	0.044*	
C111	0.54017 (18)	0.6141 (2)	0.61825 (15)	0.0129 (5)	
C112	0.56717 (19)	0.6087 (2)	0.70644 (16)	0.0174 (5)	
H112	0.6316	0.6437	0.7283	0.021*	
C113	0.50063 (19)	0.5526 (2)	0.76277 (16)	0.0177 (5)	
H113	0.5191	0.5486	0.8231	0.021*	
C114	0.40750 (18)	0.5026 (2)	0.73001 (15)	0.0150 (5)	
C115	0.37692 (18)	0.5094 (2)	0.64311 (15)	0.0151 (5)	
H115	0.3113	0.4764	0.622	0.018*	
C116	0.44426 (18)	0.5657 (2)	0.58699 (15)	0.0149 (5)	
H116	0.4246	0.571	0.5269	0.018*	
C211	0.97726 (17)	0.6546 (2)	0.59351 (14)	0.0127 (5)	
C212	0.95377 (18)	0.7715 (2)	0.59664 (15)	0.0150 (5)	
H212	0.889	0.7959	0.6213	0.018*	
C213	1.02240 (18)	0.8536 (2)	0.56474 (15)	0.0157 (5)	
H213	1.0056	0.9337	0.5672	0.019*	
C214	1.11628 (18)	0.8159 (2)	0.52909 (15)	0.0168 (5)	
C215	1.14142 (19)	0.6996 (2)	0.52416 (16)	0.0187 (6)	
H215	1.2062	0.6756	0.4994	0.022*	
C216	1.07162 (18)	0.6190 (2)	0.55558 (16)	0.0159 (5)	
H216	1.0877	0.5389	0.5515	0.019*	
N1	0.61776 (14)	0.65887 (19)	0.56113 (12)	0.0133 (4)	
N2	0.90082 (15)	0.57349 (18)	0.62280 (13)	0.0130 (4)	
O1	0.80748 (12)	0.65429 (16)	0.46501 (10)	0.0152 (4)	
O2	0.70804 (13)	0.44317 (15)	0.61821 (11)	0.0166 (4)	
Cu1	0.75794 (2)	0.58295 (3)	0.567779 (18)	0.01230 (8)	
Br1	0.32073 (2)	0.41819 (2)	0.806750 (16)	0.02103 (8)	
Br2	1.21072 (2)	0.92786 (3)	0.48364 (2)	0.03221 (9)	

### Atomic displacement parameters (Å^2^)

**Table:**

	*U*^11^	*U*^22^	*U*^33^	*U*^12^	*U*^13^	*U*^23^
C11	0.0233 (13)	0.0226 (16)	0.0175 (12)	−0.0046 (11)	−0.0012 (10)	0.0066 (11)
C12	0.0180 (11)	0.0132 (13)	0.0130 (11)	−0.0037 (10)	−0.0052 (9)	0.0005 (10)
C13	0.0199 (12)	0.0158 (14)	0.0164 (12)	−0.0007 (10)	−0.0039 (10)	0.0069 (11)
C14	0.0161 (11)	0.0154 (14)	0.0183 (12)	−0.0011 (10)	−0.0050 (9)	−0.0016 (11)
C15	0.0200 (13)	0.0201 (16)	0.0324 (15)	0.0052 (11)	−0.0024 (11)	0.0068 (13)
C21	0.0281 (14)	0.0198 (16)	0.0287 (15)	−0.0056 (12)	−0.0016 (12)	0.0103 (13)
C22	0.0205 (12)	0.0140 (14)	0.0195 (13)	0.0000 (10)	0.0036 (10)	0.0017 (11)
C23	0.0240 (13)	0.0172 (14)	0.0190 (13)	0.0008 (11)	−0.0038 (10)	0.0098 (11)
C24	0.0189 (12)	0.0185 (15)	0.0156 (12)	0.0012 (11)	−0.0008 (9)	0.0026 (11)
C25	0.0215 (13)	0.037 (2)	0.0291 (15)	−0.0055 (13)	−0.0112 (11)	0.0184 (14)
C111	0.0132 (11)	0.0131 (13)	0.0123 (11)	0.0010 (9)	−0.0011 (9)	0.0007 (10)
C112	0.0162 (12)	0.0184 (14)	0.0171 (12)	−0.0017 (10)	−0.0040 (9)	−0.0022 (11)
C113	0.0192 (12)	0.0214 (15)	0.0121 (12)	−0.0018 (11)	−0.0024 (9)	−0.0025 (11)
C114	0.0136 (11)	0.0154 (14)	0.0164 (12)	0.0025 (10)	0.0047 (9)	−0.0021 (10)
C115	0.0120 (10)	0.0166 (14)	0.0166 (12)	−0.0004 (10)	−0.0010 (9)	−0.0038 (11)
C116	0.0142 (11)	0.0167 (14)	0.0136 (11)	0.0012 (10)	−0.0014 (9)	−0.0026 (10)
C211	0.0107 (10)	0.0167 (14)	0.0103 (11)	−0.0009 (10)	−0.0032 (8)	0.0000 (10)
C212	0.0139 (11)	0.0170 (14)	0.0140 (11)	0.0015 (10)	0.0010 (9)	−0.0030 (10)
C213	0.0176 (11)	0.0121 (13)	0.0172 (12)	0.0015 (10)	−0.0013 (9)	−0.0012 (10)
C214	0.0134 (11)	0.0190 (14)	0.0182 (12)	−0.0029 (10)	0.0011 (9)	0.0039 (11)
C215	0.0144 (11)	0.0210 (15)	0.0210 (13)	0.0015 (10)	0.0049 (10)	0.0030 (11)
C216	0.0167 (12)	0.0120 (13)	0.0188 (12)	0.0023 (10)	−0.0006 (10)	−0.0009 (10)
N1	0.0118 (9)	0.0144 (11)	0.0133 (10)	−0.0015 (8)	−0.0032 (7)	0.0014 (9)
N2	0.0117 (9)	0.0126 (11)	0.0147 (10)	−0.0002 (8)	−0.0004 (7)	0.0017 (9)
O1	0.0176 (8)	0.0154 (10)	0.0123 (8)	0.0008 (7)	−0.0018 (6)	0.0021 (7)
O2	0.0172 (8)	0.0118 (10)	0.0210 (9)	−0.0019 (7)	0.0013 (7)	0.0044 (7)
Cu1	0.01157 (14)	0.01196 (17)	0.01323 (15)	−0.00026 (11)	−0.00136 (11)	0.00234 (12)
Br1	0.02047 (14)	0.02328 (16)	0.01966 (14)	−0.00309 (11)	0.00480 (10)	0.00181 (11)
Br2	0.02029 (14)	0.02105 (17)	0.0562 (2)	−0.00094 (12)	0.01245 (13)	0.01269 (15)

### Geometric parameters (Å, º)

**Table:**

C11—C12	1.509 (3)	C111—C116	1.391 (3)
C11—H11A	0.98	C111—N1	1.432 (3)
C11—H11B	0.98	C112—C113	1.387 (4)
C11—H11C	0.98	C112—H112	0.95
C12—O1	1.289 (3)	C113—C114	1.376 (3)
C12—C13	1.374 (3)	C113—H113	0.95
C13—C14	1.426 (3)	C114—C115	1.380 (3)
C13—H13	0.95	C114—Br1	1.905 (2)
C14—N1	1.321 (3)	C115—C116	1.392 (3)
C14—C15	1.505 (3)	C115—H115	0.95
C15—H15A	0.98	C116—H116	0.95
C15—H15B	0.98	C211—C212	1.385 (4)
C15—H15C	0.98	C211—C216	1.398 (3)
C21—C22	1.503 (4)	C211—N2	1.424 (3)
C21—H21A	0.98	C212—C213	1.382 (3)
C21—H21B	0.98	C212—H212	0.95
C21—H21C	0.98	C213—C214	1.385 (3)
C22—O2	1.287 (3)	C213—H213	0.95
C22—C23	1.380 (3)	C214—C215	1.383 (4)
C23—C24	1.422 (4)	C214—Br2	1.904 (3)
C23—H23	0.95	C215—C216	1.377 (4)
C24—N2	1.320 (3)	C215—H215	0.95
C24—C25	1.512 (3)	C216—H216	0.95
C25—H25A	0.98	N1—Cu1	1.958 (2)
C25—H25B	0.98	N2—Cu1	1.948 (2)
C25—H25C	0.98	O1—Cu1	1.9110 (17)
C111—C112	1.389 (3)	O2—Cu1	1.9085 (19)
			
C12—C11—H11A	109.5	C113—C112—H112	119.8
C12—C11—H11B	109.5	C111—C112—H112	119.8
H11A—C11—H11B	109.5	C114—C113—C112	119.1 (2)
C12—C11—H11C	109.5	C114—C113—H113	120.5
H11A—C11—H11C	109.5	C112—C113—H113	120.5
H11B—C11—H11C	109.5	C113—C114—C115	121.9 (2)
O1—C12—C13	125.2 (2)	C113—C114—Br1	118.74 (18)
O1—C12—C11	114.5 (2)	C115—C114—Br1	119.31 (18)
C13—C12—C11	120.3 (2)	C114—C115—C116	118.6 (2)
C12—C13—C14	127.2 (2)	C114—C115—H115	120.7
C12—C13—H13	116.4	C116—C115—H115	120.7
C14—C13—H13	116.4	C111—C116—C115	120.4 (2)
N1—C14—C13	122.4 (2)	C111—C116—H116	119.8
N1—C14—C15	121.5 (2)	C115—C116—H116	119.8
C13—C14—C15	116.2 (2)	C212—C211—C216	119.0 (2)
C14—C15—H15A	109.5	C212—C211—N2	119.1 (2)
C14—C15—H15B	109.5	C216—C211—N2	121.8 (2)
H15A—C15—H15B	109.5	C213—C212—C211	121.5 (2)
C14—C15—H15C	109.5	C213—C212—H212	119.3
H15A—C15—H15C	109.5	C211—C212—H212	119.3
H15B—C15—H15C	109.5	C212—C213—C214	118.2 (2)
C22—C21—H21A	109.5	C212—C213—H213	120.9
C22—C21—H21B	109.5	C214—C213—H213	120.9
H21A—C21—H21B	109.5	C215—C214—C213	121.7 (2)
C22—C21—H21C	109.5	C215—C214—Br2	119.67 (18)
H21A—C21—H21C	109.5	C213—C214—Br2	118.7 (2)
H21B—C21—H21C	109.5	C216—C215—C214	119.3 (2)
O2—C22—C23	125.3 (2)	C216—C215—H215	120.3
O2—C22—C21	114.8 (2)	C214—C215—H215	120.3
C23—C22—C21	119.8 (2)	C215—C216—C211	120.3 (3)
C22—C23—C24	126.5 (2)	C215—C216—H216	119.8
C22—C23—H23	116.8	C211—C216—H216	119.8
C24—C23—H23	116.8	C14—N1—C111	120.6 (2)
N2—C24—C23	123.0 (2)	C14—N1—Cu1	123.75 (16)
N2—C24—C25	120.7 (2)	C111—N1—Cu1	115.60 (16)
C23—C24—C25	116.2 (2)	C24—N2—C211	119.9 (2)
C24—C25—H25A	109.5	C24—N2—Cu1	124.07 (16)
C24—C25—H25B	109.5	C211—N2—Cu1	116.06 (15)
H25A—C25—H25B	109.5	C12—O1—Cu1	123.98 (15)
C24—C25—H25C	109.5	C22—O2—Cu1	125.06 (16)
H25A—C25—H25C	109.5	O2—Cu1—O1	145.59 (8)
H25B—C25—H25C	109.5	O2—Cu1—N2	94.80 (8)
C112—C111—C116	119.5 (2)	O1—Cu1—N2	93.64 (8)
C112—C111—N1	118.3 (2)	O2—Cu1—N1	95.43 (8)
C116—C111—N1	121.8 (2)	O1—Cu1—N1	94.70 (8)
C113—C112—C111	120.4 (2)	N2—Cu1—N1	148.20 (9)

### Hydrogen-bond geometry (Å, º)

**Table:**

*D*—H···*A*	*D*—H	H···*A*	*D*···*A*	*D*—H···*A*
C115—H115···O1^i^	0.95	2.47	3.370 (3)	157
C215—H215···O2^ii^	0.95	2.54	3.378 (3)	147

Symmetry codes: (i) −*x*+1, −*y*+1, −*z*+1; (ii) −*x*+2, −*y*+1, −*z*+1.
